# Using cost-effectiveness analyses to inform policy: the case of antiretroviral therapy in Thailand

**DOI:** 10.1186/1478-7547-4-21

**Published:** 2006-12-30

**Authors:** Sripen Tantivess, Gill Walt

**Affiliations:** 1 International Health Policy Programme, Bureau of Policy and Strategy, Ministry of Public Health, Nonthaburi, Thailand; 2 London School of Hygiene and Tropical Medicine, London, UK

## Abstract

**Background:**

Much emphasis is put on providing evidence to assist policymakers in priority setting and investment decisions. Assessing the cost-effectiveness of interventions is one technique used by policymakers in their decisions around the allocation of scarce resources. However, even where such evidence is available, other considerations may also be taken into account, and even over-ride technical evidence. Antiretroviral therapy (ART) is the most effective intervention to reduce HIV-related morbidity and prolong mortality. However, treatment provision in the developing world has been hindered by the high costs of services and drugs, casting doubts on its cost-effectiveness. This paper looks at Thailand's publicly-funded antiretroviral initiative which was first introduced in 1992, and explores the extent to which cost-effectiveness evidence influenced policy.

**Methods:**

This article reviews the development of the national ART programme in Thailand between 1992 and 2004. It examines the roles of cost-effectiveness information in treatment policy decisions. Qualitative approaches including document analysis and interview of key informants were employed.

**Results:**

Two significant policy shifts have been observed in government-organised ART provision. In 1996, service-based therapy for a few was replaced by a research network to support clinical assessments of antiretroviral medication in public hospitals. This decision was taken after a domestic study illustrated the unaffordable fiscal burden and inefficient use of resources in provision of ART. The numbers of treatment recipients was maintained at 2,000 per year throughout the 1990s. It was not until 2001 that a new government pledged to extend the numbers receiving the service, as part of its commitment to universal coverage. Several elements played a role in this decision: new groups of dominant actors, drug price reductions, a pro-active civil society movement, lessons from experience on treatment benefits, and global treatment advocacy. Unlike previous policy discourse, human rights, ethics and equity notions were explicitly raised to support therapy extension.

**Conclusion:**

In the early decision, moving from a relatively limited ART service to a research network was clearly influenced by cost-effectiveness data. But in the 2001 decision to include ART in the universal coverage package, cost-effectiveness arguments were over-ruled by other considerations. Thai ART policy was shaped by many factors, and was not a simple rational process which relied on evidence.

## Background

While human immune-deficiency virus (HIV) infection is incurable, use of antiretrovirals (ARVs) is the most effective intervention to prolong patients' lives. Combination antiretroviral therapy (ART), widely introduced in developed countries since the mid-1990s, has resulted in a dramatic decline in opportunistic diseases and therefore HIV-related mortality [1,2]. Furthermore, treated patients regain close-to-normal quality of life including working capacity [3]. Given that HIV largely affects younger-middle aged adults, expanding treatment access would prevent breakdown of societal structure and restore economic development.

Despite potentially desirable outcomes, ART provision in developing countries is limited: by the end of 2005 only 20% of the global population in need had accessed to therapy [4]. Scarce resources are crucial impediments, hindering treatment expansion. The introduction of ARVs and related services in poor settings requires not only long-term financial commitment to accommodate recurrent costs, but also the investment in health service infrastructure and workforce [5]. Given that there are many essential interventions competing for the same budget, policy makers are faced with difficult decisions in priority setting and resource allocation across health programmes.

While policy decisions in the developing world's health systems have been enhanced by the increasing availability of findings from research studies [6], these are still limited. In the case of HIV, the lack of current, setting-specific economic evaluations of existing interventions may have impeded decision-making, especially in poor countries where the efficient use of scarce resources is crucially needed [7]. Restricted ART provision in these settings is one of the reasons for the difficulties in conducting cost-effectiveness studies of treatment in the early stage of the epidemic. However, while the lack of economic analysis may be an obstacle in decision making, in practice policies can, and often are, made without this sort of evidence.

This paper describes the development of the publicly-subsidised ART programme in Thailand, and examines the role of cost-effectiveness information and other sorts of evidence in policy making. It draws on a larger research study undertaken by the first author as a PhD thesis [8]. Document review and analysis was a key approach for gathering the information on the context and evolution of the treatment programme and the introduction of research-based treatment – the so-called the Clinical Research Network – during 1996 to 1999. Government documents and evaluations as well as independent research reports and media articles provided background data. Information gathered was tested and triangulated through semi-structured interviews and participant observation at both national and provincial levels. Interviewees were identified through initial document analysis and then using snowball techniques. All the key politicians (including the Minister of Health) and senior bureaucrats involved in the policy process on ARV were interviewed, as well as others identified as relevant to the policy processes, including HIV experts and representatives of civil society organisations. In all 40 individuals were interviewed at the national level. Most interviews were taped, transcribed and then subjected to content analysis.

## International movement for ART scaling up in poor countries

Although ART had been adopted as standard care for AIDS patients in industrialised societies in the late 1990s, it remained inaccessible among most PLWHA in poor settings. In 1996 Brazil became the first developing country which provided universal coverage for ARV-based medication [9]. Following Brazil, pilot treatment programmes were initiated in several countries between the late 1990s and early 2002 [10-13]. Despite achievement in ART extension in some Latin American and the Caribbean nations [14], treatment coverage increased only slowly in most parts of the developing world, where the demand was overwhelming [4]. The high prices of the drugs and resource scarcity were two factors contributing to such limited development in treatment scaling up.

Global agencies were key players in improving access to HIV treatment and care including ART, for example the establishment of the Joint United Nations Programme on HIV/AIDS (UNAIDS)' Drug Access Initiative in 1997; and Accelerating Access Initiative in 2000 [15,16]. Furthermore, small-scale and research-based ART schemes were implemented by academic and medical institutes, donors, regional and national governments, private business, and NGOs including faith-based communities in many Latin American and African countries.

The scale up of ARV medication in poor settings was facilitated after the instigation of the Global Fund to Fight AIDS, Tuberculosis, and Malaria (GFATM) in 2002, and the launch of WHO's policy in 2003 to get 3 million PLWHA on treatment by 2005 – the so-called 3 by 5 initiative [17]. During the same period many HIV treatment schemes were instigated through organizations such as the US President's Emergency Plan for AIDS Relief (PEPFAR) and the Clinton and Bill and Melinda Gates Foundations [4]. Although the 3 by 5 target was not achieved treatment coverage in the developing world was extended significantly, from only 2% of the population in need in 2001 to 15% in 2005 [4].

## ART in Thai context: treatment programme evolution

HIV in Thailand moved rapidly from a concentrated epidemic in the early 1980s among high-risk groups such as male homosexuals, intravenous drug users and commercial sex workers, to a generalised epidemic in the beginning of 1990s [18]. Owing to effective prevention programmes such as condom promotion, public information and targeted education, the number of new infections fell dramatically from the mid-1990s. Much of Thailand's success in slowing the HIV/AIDS epidemic has been attributed to the open policy environment which promoted these programmes. Despite this declining trend, in 2005 the estimated numbers of HIV-afflicted persons and AIDS cases were 600,000 and 70,000, respectively [19]. These projections of HIV/AIDS cases included the assumption that people's risky behaviour and the Thai government policy on HIV prevention and treatment would not change from the baseline, of 2001 – the year these projections were carried out. Note that at that time ART access under the national programme was still limited.

**Figure 1:**
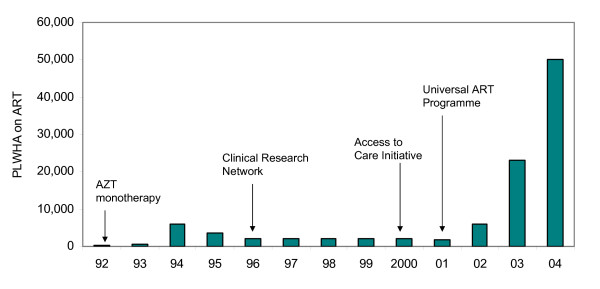
Thailand's national ART programme development, 1992–2004. Source: Bureau of AIDS, Tuberculosis and Sexually-transmitted Infections.

The national response to the disease evolved over time, and initial efforts focused on prevention. But in 1992, a limited, public-sector ART programme was implemented, offering zidovudine (AZT) monotherapy to people living with HIV/AIDS (PLWHA) in low-income groups [20]. The number of patients enrolled in this programme rose from 350 in the first year to 3,600 in 1995. See figure 1. This publicly-funded ART programme was terminated in 1996 because of concerns about quality of care and costs [20], and the Health Ministry reallocated its ART budget to what was called the Clinical Research Network – shifting the focus from treatment to the evaluation and monitoring of treatment. Fifty hospitals in Bangkok and other provinces were selected to assess the effectiveness and safety of dual-ARV combinations containing zidovudine, didanosine and zalcitabine. In 2000, the Clinical Research Network was reformulated into the Access to Care Initiative, instigated to provide highly active antiretroviral therapy (HAART) on a service basis [21]. Drug trials and operational studies under the Clinical Research Initiative provided not only empirical evidence on clinical outcomes of tested ART protocols, but also the information on treatment and care delivery for PLWHA in peripheral settings, which was helpful to the development of HIV services in later phases [22]. However, throughout the 1996 to 2001 period, the numbers of ART beneficiaries were maintained at 2,000 per annum, or approximately 3% to 6% of AIDS-afflicted population. It is noteworthy that when the Access to Care programme was instigated, there was no significant campaign for treatment extension in the public sector. At that time, the very high prices of ARVs limited coverage of the public ART services, a factor understood by NGOs and other treatment advocates [8]. The ART programme managers and HIV experts considered that international treatment guidelines had shifted from dual-therapy to HAART, and Thailand had gained considerable experience on treatment provision from the previous phase. In the absence of political pressure, the MoPH's AIDS Division set up the annual targets of this service-based initiative according to the resources available.

A dramatic policy shift took place in late-2001 as the newly elected government pledged to provide free treatment to all clinically eligible people living with HIV/AIDS (PLWHA) through the recently instigated Universal Health Coverage (UC) plan [23]. Thereafter, treatment targets escalated, from 6,500 in 2002 to 23,000 and 50,000 in 2003 and 2004, respectively. ART continued to be financed from the Health Ministry budget until it was integrated into the UC benefit package in 2005 [24].

In the following section, we analyse how far cost-effectiveness information and other contributing factors influenced policy over two periods: the replacement of limited ARV monotherapy service with the collaborative Clinical Research Network in 1996, and the adoption of universal ART policy in 2001.

## The establishment of the Clinical Research Network

Within four years after AZT was introduced, evidence from developed countries suggested limited clinical benefits from single-drug regimens. By the mid 1990s, demand for therapy in Thailand had risen sharply [20]. Policy makers in the Ministry of Health were faced with two questions: would publicly-subsidised ART offer value for money, and to what extent could the public sector afford the escalating programme costs? In 1994 the Health Ministry requested technical support from the World Health Organisation (WHO) and the World Bank to conduct a review of the existing treatment policies, as well as to examine the efficiency and affordability of possible alternatives. Based on the projections of HIV distribution in the Thai population and the costs and clinical outcomes of different treatment and prevention interventions, the study suggested that the use of ARVs available in the country, including AZT, didanosine and zalcitabine, for therapeutic purpose was considerably less cost-effective than the introduction of AZT prophylaxis to reduce mother-to-child HIV transmission [25]. For instance, the effectiveness to cost ratio of administering ARV in AIDS cases was 30 QALYs per million baht, while the corresponding ratio in the case of AZT plus breast-milk substitute was more than 600 QALYs per million baht [25:312]. Moreover, the fiscal assessment indicated that universally providing ARVs for therapeutic purposes would be unaffordable, as the financial requirements in such cases ranged from 2 to 6 times the total HIV budget. In contrast, providing AZT and infant formula to prevent vertical transmission in all HIV-positive mothers would consume only 16% of the total HIV budget.

Senior health officials noted that, in response to the study's findings, the Health Ministry had two options: to terminate ARV supply of AZT, didanosine and zalcitabine to hospitals or to improve the efficiency of the treatment programme [20]. The shift in the national ART policy from a service-based programme to the more selective Clinical Research Network of 50 hospitals in 1996 was influenced by the findings of the study, which suggested that providing universal coverage of anti-retroviral medicines would not be financially feasible. At the same time, the increase in AIDS cases was considered an opportunity to assess more systematically the effectiveness and safety of different ARV protocols (which were moving from monotherapy to combination therapies), as well as to identify appropriate regimens for use in the country.

In this shift in policy empirical information derived from programme reviews, epidemiological projections and cost-effectiveness analyses [20,25] had a crucial role in the reallocation of HIV budget. It could be argued that, however, such evidence might have been generated to justify the decision to terminate the service-based ART initiative. Implementing the clinical research project would have been an acceptable policy option to maintain treatment delivery to a limited number of patients. According to AIDS officials, even before commissioning the programme evaluation, the Health Ministry had realised that public finances would be unable to meet the growing demand for ARVs:

*'In the first years of fully subsidised ARV supply, physicians and hospital administrators were interested in HIV/AIDS care. One of the reasons was they received ARVs without any need to negotiate for more budget. ... From 1994, the MoPH's AIDS Division reached its full capacity of supply because of overwhelming requests.' *[20:431]

It is noteworthy that in 1994 the coverage of ART offered through the public initiative was only 6% of the population in need. Projecting the fiscal burden of providing universal access to treatment was thus compelling evidence of the unaffordability of programme costs [20]. On the other hand, the MoPH was in a difficult position, explaining any discontinuation of ARV services. Establishing the Clinical Research Network met the need to improve the quality of care being provided, at the same time as limiting the numbers treated in the public health system. It could be said this re-focus of the policy was skilfully handled, and there was no significant negative reaction from civil society groups.

## The adoption of universal ART policy

In 2001 the new government introduced, as part of pre-election promises, a reform of health care financing, instigating the universal health coverage plan (UC), which aimed to ensure equitable access to essential health services among Thai people [26]. Given the high costs of HAART regimens, including HAART in UC was a significant challenge, since it meant that medication coverage needed to be scaled up over 30 times in order to meet the existing needs of 60,000 AIDS-afflicted individuals. This number was expected to rise over time owing to the growing number of full-blown HIV cases, but also to the increase in patient survival due to improved ART access.

Between February and October 2001, there were intense campaigns run by ART advocate coalitions, which included, for example 53 PLWHA organizations under the Thai Network of People Living with HIV/AIDS; the National AIDS NGO Network; Drug Study Group; the Thai AIDS Society; the Thai Lawyers Association; individual scientists from the Government Pharmaceutical Organization; experts on intellectual property laws; and HIV clinicians from medical institutes [8]. While NGOs and PLWHA networks played an explicit role in organizing public fora and communicating with the Health Minister and respective health officials, other organizations provided technical and information support on clinical outcomes, prices, generic availability, patent status of ARVs, and related regulations. The discourse among these groups pointed to the clinical benefits of therapy in foreign countries and Thai settings, as well as emphasising ideals such as human rights, ethics and equity [27,28]. Re-assessing the costs and consequences of medication were among the issues raised by NGO leaders among others [8], because of rapid changes in medicines available, decreasing prices, and demonstrable successes in treatment in countries such as Brazil. Some HIV specialists also encouraged the MoPH to undertake economic appraisals of ARV medication, comparing such interventions with those for chronic conditions such as diabetes and cardiovascular diseases, which *were *subsidised by the UC plan, since they anticipated that ART administration would be more cost-effective [29]. Despite these suggestions, no economic evaluation of therapy was carried out in Thai context before the policy change.

An analysis of ART policy process in this crucial period between March and November 2001 suggests that apart from the intense domestic treatment advocacy by civil society organisations, programme expansion was facilitated by several other factors discussed in greater detail in Tantivess [8]. Among these were: changes in the administration; involvement of new actors (such as the new Health Minister, health system reformists, NGOs and PLWHA coalitions); a relatively well-developed health delivery system; lessons learned from small-scale treatment initiatives; and the global campaign to promote ART access in resource-poor countries, and generic ARV production by the government laboratory and subsequent price reduction [8]. Such elements together legitimised the shift in policy by enhancing the affordability, feasibility, and political desirability of universal ART provision through public health services. Of these, the substantial decrease in drug prices owing to local generic production was critical. NGO-PLWHA networks made use of such information to encourage the MoPH to extend treatment to cover all people in need, as doing so would become affordable. Policy discussion took place in the context of health care financing reforms, which facilitated the proposal for policy changes. The UC introduction and ART scale up shared the underlying ideal that it was people's right to access all essential health services.

## Discussion and conclusion

Policy making in HIV is complex because the causes, manifestations and implications of the epidemic are multi-dimensional, involving socioeconomic, political and health factors. The policy environment has also been extremely dynamic, as knowledge has advanced since the early 1980s. Since most HIV-afflicted people are marginalised and vulnerable to deprivation, the extent to which they can access proper care and treatment largely relies on public services [30]. To address these challenges, employing only efficiency-directed cost-effectiveness analysis in assessing the values of HIV interventions is inadequate. Laxminarayan and colleagues [31:1197] have argued *'Cost-effectiveness is only one consideration in allocating resources to specific diseases and interventions; epidemiological, medical, political, ethical, cultural, and budgetary factors also affect such decisions.'*

Economic evaluations suggest that ART in poor countries does not offer value for money since therapy is less cost-effective than HIV prevention interventions [32,33]. Following Creese and colleagues [32], the cost per DALY gained in ART introduction in African settings is approximately US$1,100–1,800, while less than US$100 is required to achieve the same benefit if particular preventive measures, such as condoms (US$1–99), peer education for commercial sex workers (US$4–7), diagnosis and treatment of sexually-transmitted infections (US$12), and voluntary counselling and HIV testing (US$18–22), is implemented. For some, this suggests that resources should be concentrated on prevention programmes rather than therapy. For others, therapy cannot be denied. Boelaert and colleagues [17] argue that the rationale for treatment provision in industrialised societies is not based on cost-effectiveness, but on its contribution to the reduction of HIV-related morbidity and mortality as well as the improved quality of life of PLWHA. In the same vein, ART scaling up in the South over recent years was not guided by results of economic evaluation. Further, existing economic appraisals are limited, usually do not capture or quantify the benefit of ARV treatment on socioeconomic development [34]. These studies therefore neglect the potential positive externalities of ART – for example encouraging people to seek counselling and testing [35].

Overall, it seems that policies to roll out ART in poor countries have been propelled by factors other than cost effectiveness considerations, and in spite of perceptions that treatment is often not cost effective. It is probably the case that in many low-income countries with high HIV prevalence, ART roll-out has been strongly influenced by the global discourse on expanding access to treatment, as well as significantly increased financial and technical support through different international initiatives [36].

In this paper we suggest that the information on cost-effectiveness played a role in the first shift in policy – with the result of limiting, rather than expanding ART. However, it could be argued that the termination of service-based therapy programme was guided by the projected financial burden of the programme, rather than the cost-effectiveness information. Indeed, the, findings from economic evaluations can be used to inform resource allocation in several ways. Although the study commissioned by the MoPH in 1995 suggested that ART for adults was less cost-effective than most HIV prevention interventions, at 33,300 baht per QALY gained, the medication could have been justified. This is because when the cost per QALY gained is compared with the national ability to pay, the benchmark usually used is the cost per QALY gained should be less than three times of gross national product [37]. Given that GNP in Thailand was 69,800 baht in 1995, some could have argued that it was therefore cost-effective to introduce ART. However, others argue that even cost-effective interventions are not always affordable. Long term concerns about the costs and affordability of ART continued to shape national treatment policies until the policy change in 2001. Clearly the dramatic decision that year to include ART in the universal coverage scheme was influenced by other factors than costs or effectiveness, with policy justification resting on ideological considerations around human rights, ethics and equity. Admittedly the considerable reduction in prices which alleviated the cost of expansion, and implicitly improved the cost-effectiveness of ARVs was also crucial in contributing to the change in policy.

In the analysis of policy, caveats are in order. This paper is based on an analysis of the policy process in the public sector, and not specifically on the attitudes of Thai policy makers towards particular techniques of resource allocation and prioritisation. Moreover, the paper focuses on ARV therapy – an intervention with unique characteristics – so the conclusions reached may not be generalisable to other policies. The medicines prescribed in ART have changed over time and while prices have fallen they remain expensive; ART is demanded by large numbers of patients and is used for an incurable disease for which prevention measures are obviously more cost-effective; ART is complicated to administer; and may cause both desirable and undesirable externalities. Finally there has been global commitment to expanding access to ARVs. These features shaped the decisions on publicly-subsidised service provision in Thailand, and may not be comparable with decision making on other health interventions. Furthermore, this study emphasises the extension of treatment delivery under the national programme organised by the Health Ministry. It excludes the adoption of ARV medication in the private sector or in the country's health benefit plans.

It is noteworthy that although the pledge to include ART in the UC benefit package was made in 2001, treatment costs were only formally covered by the Plan in 2005. This was because negotiations were necessary between the MoPH's Disease Control Department, which had been responsible for treatment provision since 1992, and the UC plan managers [8]. The transfer of programme supervision and ARV procurement roles as well as corresponding budgets was protracted and took time to resolve. Meanwhile, NGO coalitions continued to advocate for ARV-based therapy as one of the UC benefits, as they believed that this would assure the sustainability of treatment provision.

In conclusion, the allocation of public resources to subsidise ART in Thailand has always been driven by policy considerations of resource availability and affordability. However, at certain points in the policy process, other motivations, specifically over human rights and equity, were extremely important in providing the impetus for radical change. The sustainability of ART programme in Thailand remains uncertain. The drug costs may escalate dramatically if viral resistance to the current first-line regimens develops among large proportions of the treated population, resulting in the rising demand for expensive, imported original ARV products. International and bilateral regulations on trade-related intellectual property rights protection are other threats. While the production of many new generic ARVs including indinavir, ritonavir and saquinavir, as well as other drugs in new dosage forms and fix-dose combinations [38] has expanded the capacity of the ART programme, there is a continuing battle over market exclusivity of pharmaceutical products as a result of Thai-US Free Trade Agreements which may threaten the price and production of these drugs in the future [39].

## Competing interests

The author(s) declare that they have no competing interests.
